# Temperature Sensitivity of ^14^N-*V* and ^15^N-*V* Ground-State Manifolds

**DOI:** 10.1103/PhysRevApplied.19.064084

**Published:** 2023-06-30

**Authors:** Sean Lourette, Andrey Jarmola, Victor M. Acosta, A. Glen Birdwell, Dmitry Budker, Marcus W. Doherty, Tony Ivanov, Vladimir S. Malinovsky

**Affiliations:** 1 Department of Physics, University of California, Berkeley, California 94720, USA; 2 DEVCOM Army Research Laboratory, Adelphi, Maryland 20783, USA; 3 Center for High Technology Materials, and Department of Physics and Astronomy, University of New Mexico, Albuquerque, New Mexico 87106, USA; 4 Johannes Gutenberg-Universität Mainz, Mainz 55128, Germany; 5 Helmholtz-Institut, GSI Helmholtzzentrum für Schwerionenforschung, Mainz 55128, Germany; 6 Department of Quantum Science & Technology, Research School of Physics, Australian National University, Canberra 2601, Australia

## Abstract

We measure electron- and nuclear-spin transition frequencies in the ground state of nitrogen-vacancy (N-*V*) centers in diamond for two nitrogen isotopes (^14^N-*V* and ^15^N-*V*) over temperatures ranging from 77 to 400 K. Measurements are performed using Ramsey interferometry and direct optical readout of the nuclear and electron spins. We extract coupling parameters *Q* (for ^14^N-*V*), *D*, *A*_‖_, *A*_⊥_, and $\gamma_{e}/\gamma_{n}$, and their temperature dependences for both isotopes. The temperature dependences of the nuclear-spin transitions within the $m_{s}=0$ spin manifold near room temperature are found to be 0.52(1) ppm/K for ^14^N-*V*(|*m_I_* = −1⟩ ↔ |*m_I_* = +1⟩) and −1.1(1) ppm/K for ^15^N-*V*(|*m_I_* = −1/2⟩ ↔ |*m_I_* = +1/2⟩). An isotopic shift in the zero-field splitting parameter *D* between ^14^N-*V* and ^15^N-*V* is measured to be ~ 120 kHz. Residual transverse magnetic fields are observed to shift the nuclear-spin transition frequencies, especially for ^15^N-*V*. We have precisely determined the set of parameters relevant for the development of nuclear-spin-based diamond quantum sensors with greatly reduced sensitivity to environmental factors.

## I. INTRODUCTION

In recent years, color centers in diamond, and, in particular, the nitrogen-vacancy (N-*V*) center, have emerged as one of the key platforms for quantum-technology applications, particularly in sensing [1]. As the technology matures, detailed knowledge of the parameters of the system and their environmental dependence become a prerequisite for development of accurate devices such as magnetometers, gyroscopes, clocks, as well as multisensors. For nitrogen-vacancy-based rotation sensing [2–4], one may use ^14^N-*V* and ^15^N-*V* centers, where using two isotopes is important for differential measurements to separate rotational, magnetic, and temperature effects [5–10].

In this work, which follows the earlier experimental studies of the temperature dependence of the ground-state zero-field splitting parameter *D* [11], the electric quadrupole hyperfine splitting parameter *Q* [12], and the theoretical analysis [13], we present a complete experimental characterization of the temperature dependence of the coupling parameters of N-*V* centers for both nitrogen isotopes (^14^N-*V* and ^15^N-*V*), including the dependence of the magnetic hyperfine coupling parameters *A*_‖_ and *A*_⊥_. We find that N-*V* parameters are generally temperature-dependent, with relative sensitivity ranging from 7 to 90 ppm/K at room temperature, depending on the parameter.

Additional findings of this work include identification of the relatively high sensitivity of ^15^N-*V* nuclear-spin levels to misalignment of the magnetic field to the N-*V* axis, as well as measurement of the isotopic shift in *D* at a level of ~ 40 ppm. The latter is important for testing the theoretical models of the system in order to attain a level of understanding necessary for accurate modeling of devices. Note that ^15^N-*V* nuclear spins were recently explored as a resource for quantum sensing not relying on microwave (MW) or radio-frequency (rf) fields in Ref. [14].

## II. THEORETICAL BACKGROUND

The Hamiltonian for the electronic ground state of ^14^N-*V* and ^15^N-*V* [15] is given by

(1) $$H=DS_{z}^{2}+QI_{z}^{2}+A_{\|}S_{z}I_{z}+\gamma_{e}B\;S_{z}-\gamma_{n}BI_{z}\;+\frac{A_{⊥}}{2}\left(S_{+}I_{-}+S_{-}I_{+}\right),$$

where *D* is the ground-state zero-field splitting parameter of the N-*V* center, *Q* is the nuclear electric quadrupole parameter (only for ^14^N-*V*), *A*_‖_ and *A*_⊥_ are the longitudinal and transverse magnetic hyperfine coupling parameters (see Table II for parameter values at 297 K), $\gamma_{e}$ is the gyromagnetic ratio of the N-*V* center (2.8033(3) MHz/G [16]), $\gamma_{n}$ is the gyromagnetic ratio of the nitrogen nuclear spin (^14^*γ_n_* = 307.59(3) Hz/G and ^15^*γ_n_* = −431.50(4) Hz/G, **B** is the external magnetic field applied along the *z* axis (N-*V* symmetry axis), and **S** and **I** are electron- and nuclear-spin operators, respectively. In Eq. (1), we neglect the transverse components of the magnetic field B=‖B‖.

**FIG. 1.**
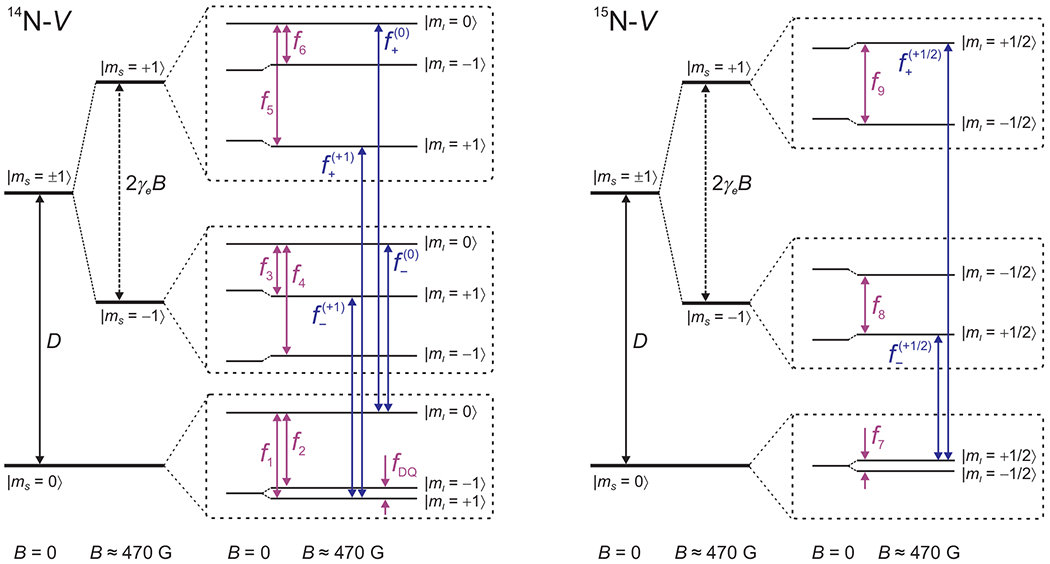
Energy-level diagrams for the electronic ground states of ^14^N-*V* and ^15^N-*V*. Energy levels are described by electron-spin (*m_s_*) and nuclear-spin (*m_I_*) quantum numbers. The electron-spin transitions used in this experiment are shown with blue arrows, and are labeled as $f_{\pm }^{\left(m_{I}\right)}$ for $\left|m_{s}=0\right\rangle ↔\left|m_{s}=\pm 1\right\rangle$, where $m_{I}$ denotes the nuclear-spin state of the transition. The nuclear-spin transitions are shown with purple arrows, and are labeled *f*_1_ to *f*_9_. The energy-level diagrams are depicted for the magnetic field values shown at the bottom.

The energy-level diagrams for the electronic ground states of ^14^N-*V* and ^15^N-*V* are shown in Fig. 1. The electron-spin transitions $\left|m_{s}=0\right\rangle ↔\left|m_{s}=\pm 1\right\rangle$ are labeled as $f_{\pm }^{\left(m_{I}\right)}$, where $m_{I}$ denotes the nuclear-spin state. The ^14^N-*V* and ^15^N-*V* nuclear-spin transitions are labeled *f*_1_ to *f*_6_ and *f*_7_ to *f*_9_, respectively, according to the diagram. The nuclear-spin double-quantum transition with frequency *f*_1_ − *f*_2_ is labeled as *f*_DQ_ and is of particular interest for rotation sensing [17,18] and comagnetometry [19,20].

Nuclear-spin transition frequencies for both ^14^N-*V* and ^15^N-*V* can be derived using perturbation theory (see Appendix C), and are described to lowest order in $A_{⊥}/\left(D\pm \gamma_{e}B\right)$ by the following expressions:

(2) f1≈|Q|+γ14nB−A⊥2D−γeB,f2≈|Q|−γ14nB−A⊥2D+γeB,f3≈|Q|−|A‖|+γ14nB,f4≈|Q|+|A‖|−γ14nB+A⊥2D−γeB,f5≈|Q|+|A‖|+γ14nB+A⊥2D+γeB,f6≈|Q|−|A‖|−γ14nB,

and

(3) f7≈|γ15n|B+A⊥22(1D−γeB−1D+γeB),f8≈A‖−|γ15n|B−A⊥22(1D−γeB),f9≈A‖+|γ15n|B−A⊥22(1D+γeB).

In this work, the transition frequencies *f*_1_ to *f*_9_, $f_{\pm }^{\left(+1\right)}$, and $f_{\pm }^{\left(+1/2\right)}$ are measured in the presence of an axial field (B≈470G) at temperatures ranging from 77 to 400 K.

## III. EXPERIMENTAL METHODS

We used a custom-built epifluorescence microscopy setup (as in Ref. [17]) to measure optically detected magnetic resonances (ODMRs) in an ensemble of N-*V* centers. Four diamond samples, whose properties are listed in Table I, were used in our experiments: two with natural isotopic ratio of nitrogen, and two with enhanced ^15^N concentration.

The diamond sample was mounted inside a continuous-flow microscopy cryostat (Janis ST-500). A bias magnetic field *B* (470–480 G) was applied along one of the N-*V* axes using two temperature-compensated samarium-cobalt (Sm_2_Co_17_ grade: EEC 2:17-TC16) ring magnets, which were arranged in a Helmholtz-like configuration that minimizes magnetic field gradients across the detected volume. An aspheric condenser lens with a numerical aperture of 0.79 was used to illuminate a ~50μm spot on the diamond with ~30mW of 532 nm laser light and collect fluorescence. The N-*V* sensing volume is ~ 10^−3^ mm^3^, defined by the area of the incident laser beam and the length of its path through the diamond. The fluorescence was separated from the excitation light by a dichroic mirror, passed through a band-pass filter (650 to 800 nm), and detected by a free-space Si photodiode. MW and rf signals were delivered using a 160-μm-diameter copper wire placed on the diamond surface next to the optical focus. The generation of MW and rf signals is described in detail in the supplemental information section of Ref. [17].

**FIG. 2.**
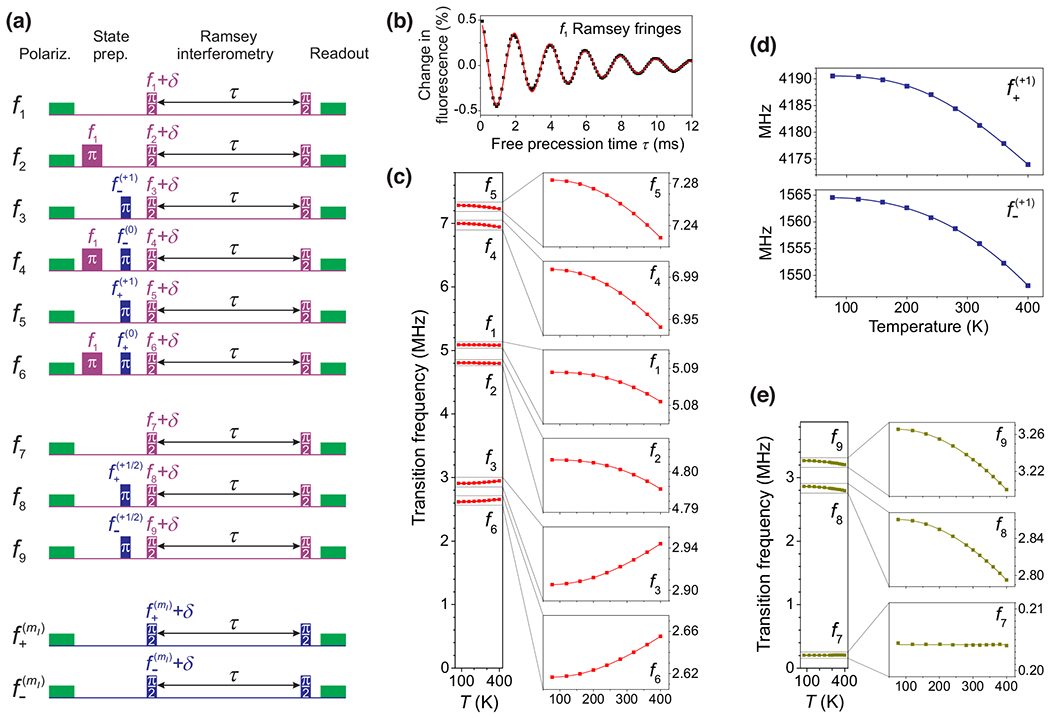
Ramsey measurements of ground-state transition frequencies. (a) Nuclear-spin transition frequencies *f*_1_ to *f*_6_ (^14^N-*V*) and *f*_7_ to *f*_9_ (^15^N-*V*) as well as electron-spin transition frequencies *f*_+_ and *f*_−_ for both isotopes are measured using Ramsey interferometry. After optical polarization (green) with green light, the nuclear spin is manipulated with a series of rf (purple) and MW (blue) pulses to prepare a superposition of the two relevant energy states. The superposition then precesses at the detuning frequency δ for a variable time τ, after which a π/2 rf pulse converts the acquired phase into a population difference to be read out optically. (b) Example of the nuclear Ramsey interferometry measurement. The oscillation frequency of the Ramsey fringes corresponds to the detuning δ from the transition frequency *f*_1_. (c) Temperature dependence of *f*_1_ to *f*_6_ for sample G2 at *B* ≈ 470 G. The *y*-axis range for the *f*_1_ and *f*_2_ subplots has been reduced (70 kHz → 20 kHz) to show the reduced temperature dependence of *f*_1_ and *f*_2_. (d) Temperature dependence of *f*_+_ and *f*_−_ for ^14^N-*V* (^15^N-*V* not shown) for sample G2 at *B* ≈ 470 G. (e) Temperature dependence of *f*_7_ to *f*_9_ for sample M1 at *B* ≈ 468 G. The *y*-axis range for the *f*_7_ subplot has been reduced (80 kHz → 12 kHz).

The transition frequencies of the N-*V* ground state were measured with Ramsey interferometry using pulse sequences that are shown in Fig. 2(a). For each nuclear-spin transition frequency (*f*) between a pair of states ($\left|m_{s},m_{I}\right\rangle$ and $\left.\left|m_{s},m_{I}^{\prime }\right\rangle \right)$), the following steps are performed: optical polarization, state preparation, Ramsey interferometry, and optical readout. Polarization is done with a green laser pulse with duration 100–200μs, which polarizes the electron and nuclear spins into |0,+1⟩ (or |0,+1/2⟩ for ^15^N-*V*). [12,21–23]. State $\left|m_{s},m_{I}\right\rangle$ is prepared by transferring population using a sequence of rf and MW π pulses. Next, a superposition of the states $\left|m_{s},m_{I}\right\rangle$ and $\left|m_{s},m_{I}^{\prime }\right\rangle$ is created using a Ramsey π/2 pulse with frequency *f*_rf_ (duration 10–100μs). The superposition then accumulates a phase at a rate given by the detuning frequency, $\delta =f_{rf}-f$ (typically 0.5–10 kHz). After a variable delay time τ, the acquired relative phase is projected into a population difference with a second π/2 pulse and read out optically. Direct optical readout of the nuclear-spin state is performed without the use of microwave mapping pulses, which is possible at magnetic fields near the excited-state level anticrossing [12]. To determine *f*, we obtain δ by fitting the Ramsey oscillations with an exponentially decaying sinusoidal function. Measurements of electron-spin transition frequencies (*f*_+_, *f*_−_) were performed in the same way, using MW pulses instead of rf pulses. Figure 2(b) shows an example of a Ramsey measurement for the nuclear transition frequency *f*_1_.

## IV. RESULTS

The transition frequencies of ^14^N-*V* (*f*_1_ to *f*_6_, $f_{+}^{\left(+1\right)}$, and $f_{-}^{\left(+1\right)}$) and ^15^N-*V* (*f*_7_ to *f*_9_, $f_{+}^{\left(+1/2\right)}$, and $f_{-}^{\left(+1/2\right)}$) were measured using the previously described Ramsey interferometry technique [Figs. 2(a) and 2(b)] in the presence of an axial magnetic field B≈470G for temperatures ranging from 77 to 400 K using four samples; see Table I. For each temperature, the system was allowed to equilibrate for several hours, after which we sequentially measured each of the transition frequencies of electron spins, then nuclear spins, and then electron spins again. This was done in order to ensure the temperature drift was sufficiently small during the ~ 30-minute measurement period.

Figures 2(c) and 2(d) show the temperature dependence of the nuclear-spin and electron-spin transitions in ^14^N-*V* for sample G2. Transition frequencies *f*_1_ and *f*_2_ are least sensitive to temperature, varying by 8 kHz across the range, while *f*_3_, *f*_6_ and *f*_4_, *f*_5_ vary by ~ 40 kHz and ~ 50 kHz, respectively. These temperature dependences are largely determined by the temperature dependence of *Q* and *A*_‖_, according to Eq. (2). Figure 2(e) shows the temperature dependence of the nuclear-spin transitions in ^15^N-*V* for sample M1, which has been isotopically enriched with ^15^N. Transition frequencies *f*_8_ and *f*_9_ were measured to vary by ~ 70 kHz across the measured range of temperatures, while the temperature dependence of *f*_7_ was found to be three orders of magnitude smaller; see Sec. V for detailed analysis.

Using all measured transition frequencies, we apply the numerical methods described in Appendix A to extract values for the coupling parameters for both ^14^N-*V* (*D*, *Q*, *A*_‖_, *A*_⊥_, and γe/γ14n) and ^15^N-*V* (*D*, *Q*, *A*_‖_, *A*_⊥_, and γe/γ15n.

**FIG. 3.**
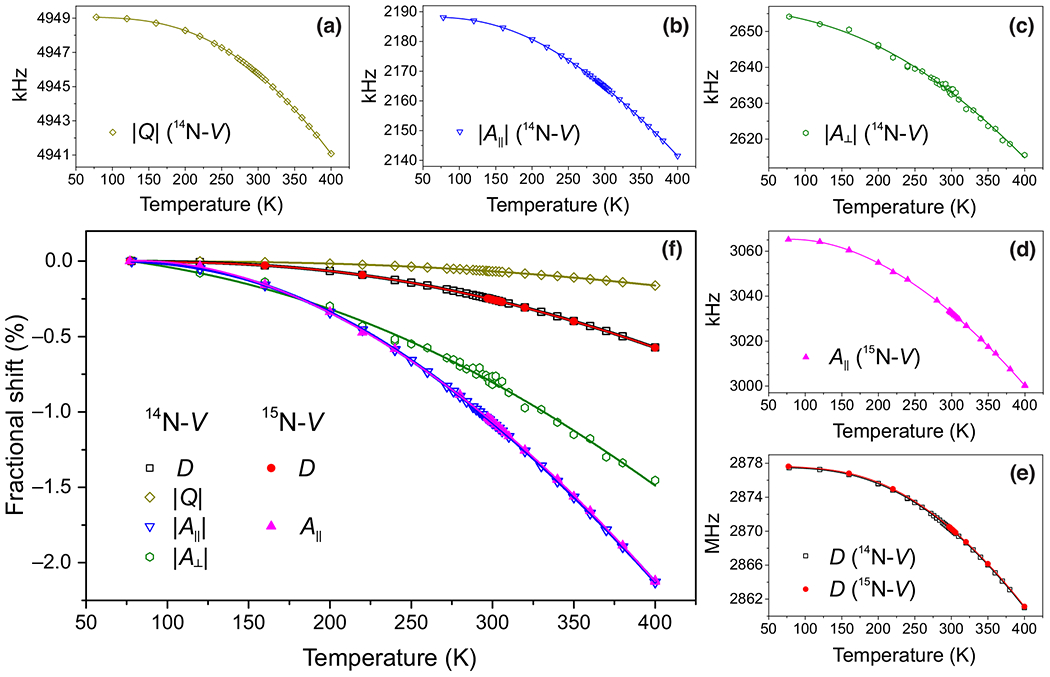
Temperature dependence of coupling parameters. (a)–(e) Coupling parameters *D*, *Q*, *A*_‖_, and *A*_⊥_ were extracted numerically (see Appendix A) from measured nuclear-spin and electron-spin transition frequencies. Coupling parameters are plotted against temperature, for both ^14^N-*V* and ^15^N-*V*, using data from all diamond samples. The solid lines are fourth-degree polynomial fits. (f) Fractional temperature dependence of all parameters whose data are presented in panels (a)–(e). In those, ^14^N-*V* and ^15^N-*V* were found to have similar fractional temperature shifts in *D* and in *A*_‖_.

Numerically extracted coupling parameters for ^14^N-*V* (*D*, *Q*, *A*_‖_, and *A*_⊥_) and ^15^N-*V* (*D* and *A*_‖_) are plotted as a function of temperature in Figs. 3(a)–3(e). Each parameter’s temperature dependence was fitted to a fourth-order polynomial. Figure 3(f) shows the fractional shift of each parameter as a function of temperature. We observe ^14^N-*V* and ^15^N-*V* to have identical fractional dependence for both *D* and *A*_‖_.

In the case of *A*_⊥_ for ^15^N-*V*, the following factors prevented the determination of its temperature dependence: (i) *A*_⊥_ has a weak contribution to the nuclear-spin transition frequencies; (ii) the ^15^N-*V* nuclear-spin transition frequencies (*f*_7_ in particular) are sensitive to magnetic field misalignment θ (see Sec. V A); and (iii) ^15^N-*V* has only three nuclear-spin transitions, whose frequencies are determined by four parameters, i.e., *A*_‖_, *A*_⊥_, γ15nB, and θ. We were able to overcome these issues at room temperature by scanning the transverse magnetic field with coils [see Fig. 5(c)] and obtained *A*_⊥_ = 3.68(2) MHz, which is in agreement with Ref. [16]. In future studies, this approach can be used to obtain the temperature dependence of ^15^N-*V A*_⊥_.

Table II lists the values and temperature derivatives of each coupling parameter at 297 K for both ^14^N-*V* and ^15^N-*V*, obtained from the polynomial fits. The room-temperature value for each measured parameter is consistent with previously reported values [16,22–24]. Temperature derivatives are also consistent with previously reported values for ^14^N-*V* parameters *D* [11,25,26], *Q* [12,26–28], and *A*_⊥_ [26–28]. Recent theoretical work [29] provided *ab initio* evaluation of the temperature dependences of several parameters for the ^14^N-*V* system. In the cases where the same parameters were calculated in Ref. [29] and measured here, we find good agreement. The temperature derivatives for ^14^N-*V A*_⊥_ and ^15^N-*V A*_‖_ are reported here for the first time.

**FIG. 4.**
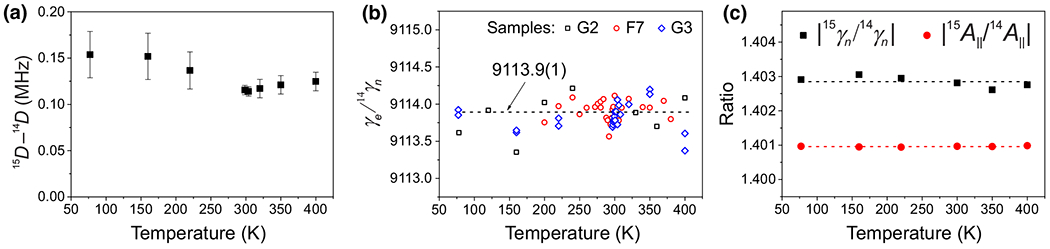
Temperature-insensitive parameters. (a) Difference in the zero-field splitting parameter *D* between ^14^N-*V* and ^15^N-*V*. (b) Ratio of electron-spin gyromagnetic ratio $\gamma_{e}$ and ^14^N-*V* nuclear-spin gyromagnetic ratio γ14n. (c) Isotopic ratios of $\gamma_{n}$ and of *A*_‖_ for ^14^N-*V* and ^15^N-*V*. Markers are experimental data, and dashed lines are mean values. In panels (a) and (c), the data were measured using sample G3, which has a 50:50 ratio of [^15^N-*V*]:[^14^N-*V*]. The error bars were determined from the reproducibility of the results.

We observe an isotopic shift of ~ 120 kHz (~ 40 ppm) in the *D* parameter, with *D* larger for ^15^N-*V*. Figure 4(a) shows the shift in *D* across the full range of measured temperatures for the G3 sample, which has a 50:50 isotopic ratio (grown with evenly mixed ^14^N and ^15^N). This effect is also clearly visible as a difference in separation of peaks in the ODMR signal (see Appendix B). The relatively small isotopic effect in *D* is in line with the fact that even a replacement of the species adjacent to the vacancy changes the zero-field splitting only slightly; see recent work [30], where the *D* values were reported to be 2888 MHz and 2913 MHz for the O-*V*^0^ and B-*V*^−^, respectively.

Figure 4(b) shows the numerically extracted values of γe/γ14n for three different samples. The mean value of γe/γ14n is measured to be 9113.9(1), which is in agreement with Ref. [31]. Using the literature value for $\gamma_{e}=2.8033(3)\;MHz/G$ [16], this corresponds to γ14n=307.59(3)Hz/G. The value for γe/γ14n can also be approximated without numerical methods from the measured frequencies directly [see Eq. (2)]:

(4) γeγ14n≈f+(0)−f−(0)f3−f6≈f+(+1)−f−(+1)+f5−f3f3−f6.

Figure 4(c) shows the isotopic ratio of γnγ15n/γ14n=1.40285(6), in agreement with Ref. [32]) and the isotopic ratio of A‖A15‖/A14‖∣=1.40096(1) obtained from measurements using the G3 sample. The difference in these ratios can be used to verify theoretical models. When using the literature value for $\gamma_{e}$, we obtain γ15n=−431.50(4)Hz/G.

## V. DISCUSSION

### A. Temperature and angular dependences of *f*_DQ_ and *f*_7_

The nuclear-spin transitions *f*_DQ_ and *f*_7_ within the $m_{s}=0$ manifold of ^14^N-*V* and ^15^N-*V* are of particular interest for sensing applications such as rotation sensing [17,18] and comagnetometry [19,20]. Precise knowledge of their transition frequencies and their dependence on environmental factors (temperature, magnetic field) is essential for optimal sensor performance. The transition frequencies *f*_DQ_ and *f*_7_ can be obtained from Eqs. (2) and (3) and are described by the following expressions:

(5) fDQ≈2×γ14nB(1−|γeγ14n|A⊥2D2−γe2B2),

(6) f7≈|γ15n|B(1+|γeγ15n|A⊥2D2−γe2B2).

The frequencies of these transitions do not depend on the coupling parameters *Q* and *A*_‖_, and are determined primarily by the nuclear Zeeman shift $\Delta m_{I}\gamma_{n}B$, resulting in a greatly reduced temperature dependence compared to other nuclear-spin transitions. Therefore, measurements of the temperature dependences of *f*_DQ_ and *f*_7_ require more precise control of the bias magnetic field.

**FIG. 5.**
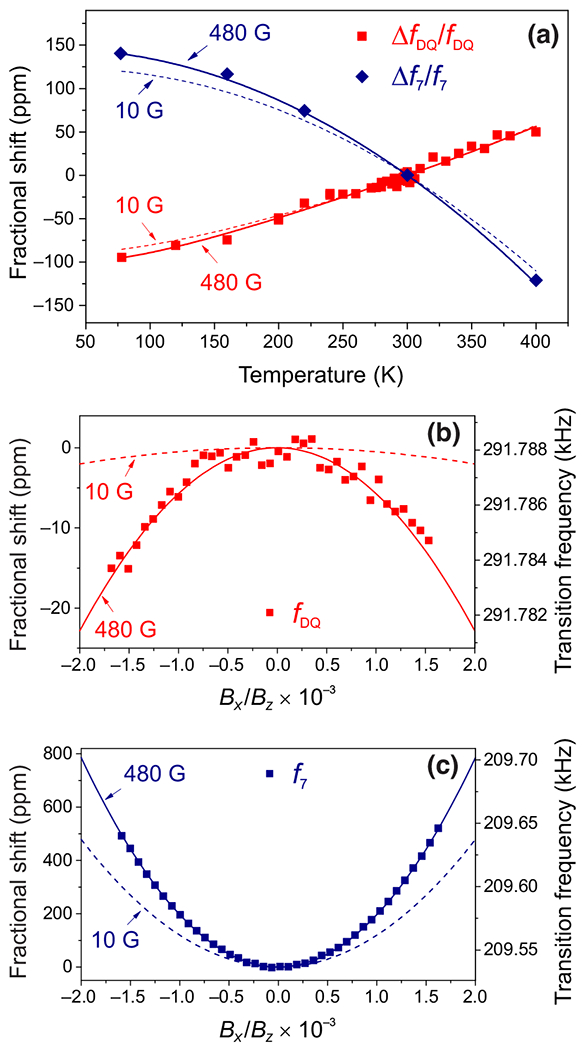
Temperature and angular dependences of *f*_DQ_ and *f*_7_. (a) The fractional shifts in nuclear transition frequencies *f*_DQ_ (red) and *f*_7_ (blue) are plotted as a function of temperature. Markers represent experimental data after correcting for variations in the magnetic field between measurements. Solid and dashed lines were obtained from Eqs. (5) and (6) at 480 G and 10 G, respectively. (b),(c) The fractional shifts in nuclear transition frequencies *f*_DQ_ (red) and *f*_7_ (blue), respectively, are plotted as a function of magnetic field misalignment with respect to the N-*V* axis for fixed values of *B_z_*. Markers represent experimental data, and solid (480 G) and dashed (10 G) lines were obtained by numerically diagonalizing the Hamiltonian [Eq. (1)] using values from Table II. For *f*_7_ in panel (c), *A*_⊥_ is treated as a free parameter, and fit to the experimental data in order to obtain *A*_⊥_ = 3.68(2) MHz.

Over the range of temperatures used in this experiment, the bias magnetic field varied by ~ 1 G, primarily due to thermal expansion of the sample holder in the presence of magnetic field gradients. This variation in the bias magnetic field was measured using electron-spin transition frequencies and subsequently used to obtain corrected values for *f*_DQ_ and *f*_7_ corresponding to 480 G. When the temperature is changed from 77 to 400 K, the corrected values of *f*_DQ_ and *f*_7_ are observed to shift by 140 ppm (44 Hz at 480 G) and −260 ppm (−55 Hz at 480 G), respectively; see Fig. 5(a). This corresponds to fractional temperature derivatives of 0.52(1) ppm/K (0.15 Hz/K) for *f*_DQ_ and −1.1(1) ppm/K (−0.10 Hz/K) for *f*_7_ at 297 K.

The temperature dependences of *f*_DQ_ and *f*_7_ arise from the temperature dependence of $A_{⊥}^{2}/D^{2}$ and are described by Eqs. (5) and (6). These equations are used with experimentally obtained polynomial fits of *A*_⊥_ and *D* (see Fig. 3) to generate the solid and dashed lines in Fig. 5(a). For *f*_7_, *A*_⊥_ is assumed to have the same fractional dependence on temperature as *A*_‖_. For sufficiently small fields $\gamma_{e}B\ll D$ (i.e., B=10G), the transition frequencies are approximately linear in the magnetic field, and thus the fractional shift is independent of magnetic field.

Magnetic field misalignment (from the N-*V* axis) is another factor that can significantly shift the transition frequencies of *f*_DQ_ and *f*_7_, and must be considered in sensing applications. Angular-dependent shifts in *f*_7_ are significantly stronger than shifts in *f*_DQ_ because of the lack of a stabilizing coupling parameter (i.e., *Q*) in the effective nuclear-spin Hamiltonian. The frequency shifts *f*_DQ_ and *f*_7_ exhibit a quadratic dependence on misalignment angle, and are shown in Figs. 5(b) and 5(c), respectively. The alignment of the magnetic field was controlled using two pairs of coils oriented perpendicular to the bias magnetic field *B_z_*. The transverse magnetic field *B_x_* was precisely determined using the N-*V* electron-spin transitions of the three nonaxial N-*V* subensembles. We measure a frequency shift of 5.0 Hz in *f*_DQ_ and of 130 Hz in *f*_7_ when misaligning the magnetic field by $\theta =0.1^{\circ }\left(B_{x}\approx 0.8\;G\right)$ at a field of $B_{z}=480\;G$.

We use numerical methods (Appendix A) together with experimental values from Table II to obtain theoretical predictions for the angular dependence of *f*_DQ_ and *f*_7_ at 480 G (solid line) and 10 G (dashed line). For *f*_7_, we fit the theoretical model to the experimental data in order to obtain a more precise value of *A*_⊥_ for ^15^N-*V*, which we measure to be 3.68(2) MHz at 297 K.

The frequency shifts in *f*_DQ_ and *f*_7_ due to magnetic field misalignment can be approximated using perturbation theory; see Appendix C. For *f*_DQ_ the dominant term is a second-order correction, whose fractional shift is described by the following expression:

(7) ΔfDQ2×γ14nBz≈12βθ2,β=−γeγ14n4|A‖|Dγe2Bz2(D2−γe2Bz2)2,

where θ is the angle between the N-*V* axis and the magnetic field, and β≈−9.9 at $B_{z}=480\;G$, and β≈−0.003 at $B_{z}=10\;G$.

While the second-order correction is similar to that of *f*_DQ_, for *f*_7_ the fourth-order correction is much larger and is described by the following expression:

(8) Δf7γ15nBz≈12βθ2,β=γe2γ15n24A⊥2D2(D2−γe2Bz2)2,

where β≈460 at $B_{z}=480\;G$, and β≈280 at $B_{z}=10\;G$.

### B. Anisotropy of the hyperfine coupling

The temperature dependence of the magnetic hyperfine coupling components, *A*_‖_ and *A*_⊥_, is used to obtain the temperature dependence of the Fermi-contact and dipolar terms. These in turn can be expressed in terms of the effective spin density η occupying the atomic orbitals of the nitrogen atom and their effective hybridization ratio ${\left|c_{p}\right|}^{2}/{\left|c_{s}\right|}^{2}$ via the expressions

(9) $$f=\frac{8\pi }{3}\frac{\mu_{0}}{4\pi }g_{e}\mu_{B}\mu_{n}{\left|c_{s}\right|}^{2}\eta {\left|\psi_{s}(0)\right|}^{2}\;=1811\;\text{MHz}\times \left(1-{\left|c_{s}\right|}^{2}\right)\eta ,$$

(10) $$d=\frac{2}{5}\frac{\mu_{0}}{4\pi }g_{e}\mu_{B}\mu_{n}{\left|c_{p}\right|}^{2}\eta \langle \psi_{p}\left|\frac{1}{r^{3}}\right|\psi_{p}\rangle \;=55.52\;\text{MHz}\times {\left|c_{p}\right|}^{2}\eta .$$

**FIG. 6.**
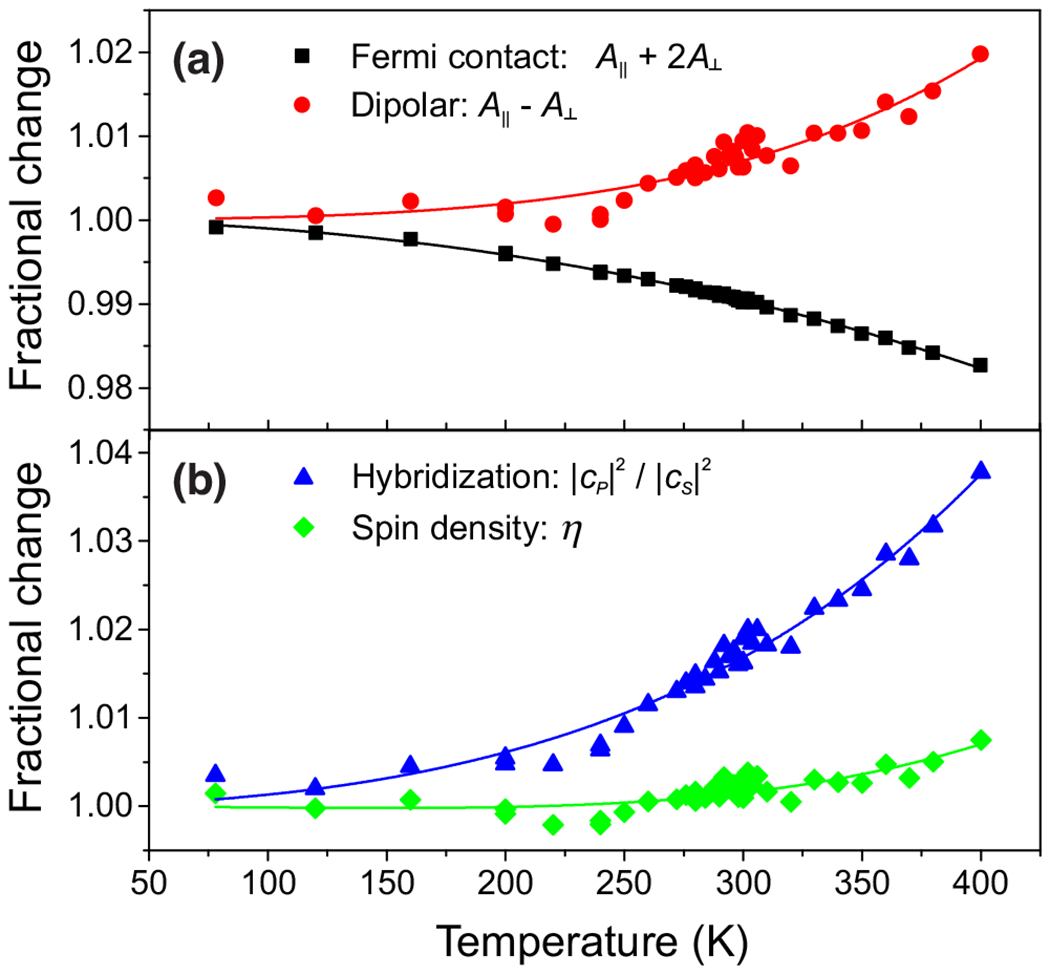
Anisotropy of the ^14^N-*V* magnetic hyperfine coupling. Temperature dependences of (a) the Fermi-contact (*f* = *A*_‖_ + 2*A*_⊥_) and dipolar (*d* = *A*_‖_ − *A*_⊥_) terms, and (b) the N-*V* orbital hybridization |*c_p_*|^2^/|*c_s_*|^2^ and spin density η obtained from Eqs. (9) and (10). Markers are experimental data, and solid lines are polynomial fits.

Figure 6 shows that both the spin density and hybridization ratio are observed to increase with temperature. This is consistent with the *ab initio* calculations [33], which concluded that, with increasing temperature, the spin density diffuses away from the three carbon atoms surrounding the vacancy and the nitrogen atom moves towards the vacancy (away from its nearest-neighbor carbon atoms). Thus, one would expect the spin density to increase at the nitrogen atom as a result of the outward diffusion, and the displacement of the nitrogen atom would lead to an increase in the hybridization ratio (as the orbitals connecting the nitrogen to its nearest neighbors must become more *p*-like to achieve the new geometry of the bond).

## VI. CONCLUSION AND OUTLOOK

We measured the nuclear-spin and electron-spin transition frequencies for N-*V* centers containing ^14^N and ^15^N, as a function of temperature. To describe the results, we used numerical diagonalization of the Hamiltonian, including the effect of magnetic field misalignment. The model allows us to extract the underlying parameters *Q* (for ^14^N-*V*, *D*, *A*_‖_, *A*_⊥_, and $\gamma_{e}/\gamma_{n}$ for both isotopes and their temperature dependences (except *A*_⊥_ for ^15^N-*V*). The magnitude of each one of these parameters (*Q*, *D*, *A*_‖_, and *A*_⊥_) decreases with temperature in the range from 77 to 400 K, showing a reduction of ~ 0.1% (in the case of *Q*) to ~ 2% (in the case of *A*_‖_).

Comparison of the determined parameters reveals a difference in *D* of ~ 120 kHz (~ 40 ppm) between N-*V* centers containing ^14^N and ^15^N. This is the first report of such an isotopic difference for N-*V* centers. We also observe a difference of ~ 0.1% between ^15^*A*_‖_/^14^*A*_‖_ and γ15n/γ14n.

The temperature dependence of the anisotropy of the hyperfine coupling between electron and nuclear spins (*A*_‖_, *A*_⊥_) in ^14^N-*V* can be used to infer the temperature dependence of the Fermi-contact and dipolar interactions, which, in turn, can provide information about the electron-spin density and orbital hybridization.

We determined the temperature dependence of *f*_DQ_ (0.52(1) ppm/K) and *f*_7_ (−1.1(1) ppm/K), which are three orders of magnitude smaller than the other nuclear-spin transition frequencies. Nevertheless, this sensitivity to temperature may limit the performance of nuclear-spin-based sensors and should be taken into account.

We found that residual transverse fields should be carefully considered in order to precisely determine the frequencies, especially for *f*_7_, for which a misalignment of the field by 0.1° leads to a fractional change of ~ 600 ppm. This strong dependence allowed us to measure *A*_⊥_ for ^15^N-*V* to be 3.68(2) MHz, which is in agreement with the previously measured value [16].

The combination *f*_3_ − *f*_6_ has negligible dependence on *A*_⊥_ and is described to high precision by $f_{3}-f_{6}=2\gamma_{n}B$, and therefore its temperature dependence is predicted to be weak: <10ppb/K.

In summary, we have precisely determined the set of parameters relevant for the development of N-*V*–diamond rotation sensors, magnetometers, frequency standards, and multisensors, along with the temperature dependence of these parameters. The results indicate a promising path to developing such devices with greatly reduced sensitivity to environmental variations. The general idea is to use multiple transitions with different sensitivity to, for example, temperature, which allows one to isolate the environmental parameter drift from the effect of interest (e.g., inertial rotation).

## Figures and Tables

**TABLE I.** Diamond samples. The estimated concentrations of substitutional nitrogen [N], N-*V* centers [N-*V*], and ^13^C atoms [^13^C], in addition to the nitrogen isotopic ratio [^14^N]:[^15^N], electron-spin dephasing time $T_{2}^{*}$, and electron-spin coherence time *T*_2_, are listed for each sample used in experiments. Millimeter-sized bulk diamonds were grown using chemical vapor deposition and were obtained from Element Six. Electron-spin $T_{2}^{*}$ was measured using Ramsey interferometry, and *T*_2_ was measured using Hahn echo techniques.

Sample	[N] (ppm)	[N-*V*] (ppm)	[^14^N]:[^15^N]	[^13^C] (%)	$T_{2}^{*}$ (μs)	*T*_2_ (μs)
F7	~ 0.1	~ 0.01	99.6:0.4	1.1	1.8(2)	600(20)
G2	10	4	99.6:0.4	<0.01	1.0(2)	11(1)
M1	~ 10	~ 1	20:80	…	0.25(2)	15(2)
G3	16	4.5	50:50	1.1	0.35(3)	22(2)

**TABLE II.** Experimentally determined coupling parameters at 297 K. The values and temperature derivatives of coupling parameters ^14^N-*V* (*D*, *Q*, *A*_‖_, and *A*_⊥_) and ^15^N-*V* (*D* and *A*_‖_) at 297 K are obtained from the polynomial fits of the temperature dependences shown in Fig. 3. The *A*_⊥_ value for ^15^N-*V* is obtained by scanning the transverse magnetic field with coils; see Fig. 5(c).

Isotope	Parameter	Value (kHz)	First derivative (Hz/K)	Fractional derivative (ppm/K)	Second derivative (Hz/K^2^)
^14^N-*V*	*D*	2870.28(3) × 10^3^	−72.5(5) × 10^3^	−25.3(2)	−0.39(1) × 10^3^
	*Q*	−4945.88(1)	35.5(3)	−7.17(6)	0.22(1)
	*A*_‖_	−2165.19(8)	197(1)	−91.0(5)	0.73(6)
	*A*_⊥_	−2635(2)	154(5)	−58(2)	0.53(3)
^15^N-*V*	*D*	2870.38(3) × 10^3^	−72(1) × 10^3^	−25.1(3)	−0.40(2) × 10^3^
	*A*_‖_	3033.3(1)	−269(3)	−89(1)	−0.98(8)
	*A*_⊥_	3680(20)			

**FIG. 7.**
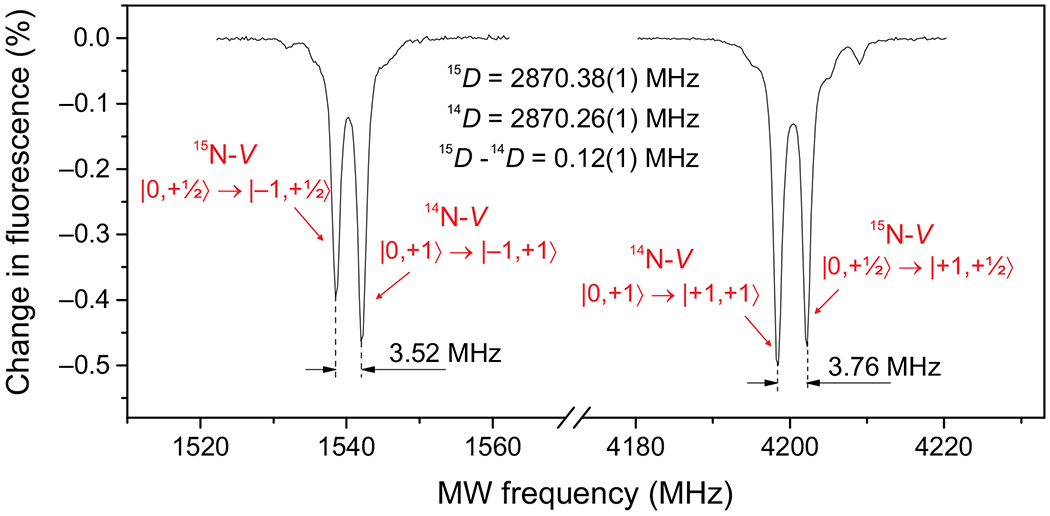
Measurement of isotopic shift in *D* using ODMR. The spectrum is obtained from the G3 sample (50:50 isotopic ratio) at 475 G and consists of four resonances corresponding to two transitions (*f*_+_, *f*_−_) for ^14^N-*V* and two transitions (*f*_+_, *f*_−_) for ^15^N-*V*. At this field, the nuclear spins are optically polarized to their largest *m_I_* sublevels, *m_I_* = +1 for ^14^N-*V* and *m_I_* = +1/2 for ^15^N-*V*, which creates a resolvable splitting. A slight difference is observed between the splitting of *f*_+_ (3.76 MHz) and that of *f*_−_ (3.52 MHz), which corresponds to a difference in *D* of 0.12(1) MHz.

## APPENDIX A: NUMERICAL METHODS – EIGENVALUES AND LEAST-SQUARES FITTING

The full ground-state Hamiltonian of ^14^N-*V* (and ^15^N-*V*, which is noted in parenthesis throughout this section) is

(A1)
$$H=DS_{z}^{2}+QI_{z}^{2}+A_{\|}S_{z}I_{z}+\gamma_{e}B_{z}S_{z}-\gamma_{n}B_{z}I_{z}\;+\frac{A_{⊥}}{2}\left(S_{+}I_{-}+S_{-}I_{+}\right)+\gamma_{e}B_{x}S_{x}-\gamma_{n}B_{x}I_{x},$$

and includes a transverse magnetic field, which is assumed without loss of generality to point in the +*x* direction. (For ^15^N-*V*, use Q=0.) Given a set of values for the coupling parameters of ^14^N-*V* (and ^15^N-*V*), which we will denote as the vector

a=〈D,γeBz,Q,A‖,A⊥,γeBx,γe/14γn,(D,A‖,A⊥,γe/γ15n)〉,

we can use eigenvalue decomposition to numerically obtain the nine (plus six) energy eigenvalues and therefore the frequencies of the six (plus three) rf transitions and two (plus two) MW transitions of the ground-state Hamiltonian of the ^14^N-*V* center, denoted as

$$f(a)=\langle f_{1},f_{2},f_{3},f_{4},f_{5},f_{6},f_{+}^{\left(+1\right)},f_{-}^{\left(+1\right)},\;\left(f_{7},f_{8},f_{9},f_{+}^{\left(+1/2\right)},f_{-}^{\left(+1/2\right)}\right)\rangle .$$

Here, we need to solve the inverse problem, starting with experimental values for the transition frequencies, **f**, and ending with values for the coupling parameters, **a**. This is done starting with an initial guess for the coupling parameters, **a**_0_, calculating the transition frequencies associated with this guess **f**(**a**_0_), and an associated weighted error between the calculated and measured transition frequencies $𝒮(a)=\sum_{i}{\left|\left(1/\delta f_{i}\right)\left(f_{i}(a)-f_{i}\right)\right|}^{2}$, where δf is the measurement uncertainty associated with **f**. The fitted values for the coupling parameters correspond to the value of **a** that minimizes 𝒮(a), which was obtained using MATLAB’s *fminsearch* function (Nelder-Mead simplex algorithm). This process was repeated for each temperature to obtain the temperature dependence of each parameter.

**TABLE III.** Transition frequencies and temperature derivatives at T=297K and B=470G. Values are determined by performing numerical diagonalization of the Hamiltonian [Eq. (1)] using values and uncertainties of *D*, *Q*, *A*_‖_, and *A*_⊥_ listed in Table II. For ^15^N-*V*, it is assumed that *A*_⊥_ has the same fractional temperature dependence as *A*_‖_ within 5%, or $\left(dA_{⊥}/dT\right)/A_{⊥}=1.00(5)\times \left(dA_{\|}/dT\right)/A_{\|}$.

Parameter	Value (kHz)	Derivative (Hz/K)
*f*_1_	5085.95(1)	−35.2(2)
*f*_2_	4799.65(1)	−35.3(2)
*f*_3_	2925.22(8)	161.5(7)
*f*_4_	6970.98(8)	−232.8(7)
*f*_5_	7257.28(8)	−232.7(7)
*f*_6_	2636.14(8)	161.5(7)
*f*_1_ − *f*_2_	286.299(2)	0.149(8)
*f*_5_ − *f*_4_	286.299(2)	0.149(8)
*f*_3_ − *f*_6_	289.081(2)	−0.000(0)
*f*_7_	205.89(3)	− 0.31(2)
*f*_8_	2825.8(1)	−268(2)
*f*_9_	3234.8(1)	−269(2)

Using the fitted temperature dependence of the coupling parameters, we numerically diagonalize the Hamiltonian at and near 297 K for B=470G. This allows us to estimate the temperature dependence of the magnetically sensitive nuclear transitions (*f*_1_ to *f*_9_), which are shown in Table III.

## APPENDIX B: ISOTOPIC SHIFT IN *D*

The isotopic shift in *D* can be measured directly from the pulsed ODMR spectrum, which is shown in Fig. 7. Signals were recorded for the G3 sample at 475 G and 298 K. At this field, the nuclear spins are optically polarized to their largest *m_I_* sublevels, *m_I_* = +1 for ^14^N-*V* and *m_I_* = +1/2 for ^15^N-*V*, which are individually resolvable due to the opposite signs of γ14n and γ15n. By extracting *D* using (*f*_+_ + *f*_−_)/2 for each isotope, we obtain *D* = 2870.26 MHz for ^14^N-*V* and *D* = 2870.38 MHz for ^15^N-*V*, which correspond to an isotopic shift in the *D* parameter of 0.12(1) MHz.

## APPENDIX C: PERTURBATION THEORY

Perturbation theory can be used to describe how both the transverse hyperfine coupling parameter (*A*_⊥_) and transverse magnetic fields (*B_x_*) shift the nuclear-spin transition frequencies for both ^14^N-*V* and ^15^N-*V*. Terms in the Hamiltonian [Eq. (A1)] can be divided into terms that do (*H*_‖_) and do not (*V*) commute with *S_z_* and *I_z_*:

(C1)
$$H_{\|}=DS_{z}^{2}+QI_{z}^{2}+A_{\|}S_{z}I_{z}+\gamma_{e}B_{z}S_{z}-\gamma_{n}B_{z}I_{z},$$

(C2)
$$V=\frac{A_{⊥}}{2}\left(S_{+}I_{-}+S_{-}I_{+}\right)+\frac{\gamma_{e}B_{x}}{2}\left(S_{+}+S_{-}\right).$$

Here we have omitted the transverse nuclear-spin Zeeman term, which is small compared to the transverse electronspin Zeeman term. Using *H*_‖_ as the unperturbed Hamiltonian and treating *V* as a perturbation, the second-order perturbation shift of each unperturbed state is calculated using the following expression:

(C3)
$$\text{Δ}E_{m_{s},m_{I}}^{\left(2\right)}=\sum_{m_{s}^{\prime },m_{I}^{\prime }}\frac{{\left|\langle m_{s}^{\prime },m_{I}^{\prime }|V|m_{s},m_{I}\rangle \right|}^{2}}{E_{m_{s},m_{I}}^{\left(0\right)}-E_{m_{s}^{\prime },m_{I}^{\prime }}^{\left(0\right)}}.$$

Here the eigenstates of the unperturbed state are denoted as $\left|m_{s},m_{I}\right\rangle$, and their energies are described as follows:

(C4)
$$E_{m_{s},m_{I}}^{\left(0\right)}=m_{s}^{2}D+m_{I}^{2}Q+m_{s}m_{I}A_{\|}+m_{s}\gamma_{e}B_{z}-m_{I}\gamma_{n}B_{z}.$$

For each nuclear-spin transition, an approximate expression for the total energy shift is obtained to second order in $1/F_{\pm }$, where $F_{\pm }=D\pm \gamma_{e}B$. There are also fourth-order perturbation shifts that produce effects that are of second order in $1/F_{\pm }$, which appear when $m_{s}^{\prime \prime }=m_{s}$ and $m_{I}^{\prime \prime }\neq m_{I}$:

(C5)
$$\text{Δ}E_{m_{s},m_{I}}^{\left(4\right)}=\sum_{m_{s}^{\prime \prime \prime },m_{I}^{\prime \prime \prime }}\sum_{m_{I}^{\prime \prime }\neq m_{I}}\sum_{m_{s}^{\prime },m_{I}^{\prime }}\frac{\langle m_{s},m_{I}|V|m_{s}^{\prime \prime \prime },m_{I}^{\prime \prime \prime }\rangle \;\langle m_{s}^{\prime \prime \prime },m_{I}^{\prime \prime \prime }|V|m_{s},m_{I}^{\prime \prime }\rangle \;\langle m_{s},m_{I}^{\prime \prime }|V|m_{s}^{\prime },m_{I}^{\prime }\rangle \;\langle m_{s}^{\prime },m_{I}^{\prime }|V|m_{s},m_{I}\rangle }{\left(E_{m_{s},m_{I}}^{\left(0\right)}-E_{m_{s}^{\prime \prime \prime },m_{I}^{\prime \prime \prime }}^{\left(0\right)}\right)\;\left(E_{m_{s},m_{I}}^{\left(0\right)}-E_{m_{s},m_{I}^{\prime \prime }}^{\left(0\right)}\right)\;\left(E_{m_{s},m_{I}}^{\left(0\right)}-E_{m_{s}^{\prime },m_{I}^{\prime }}^{\left(0\right)}\right)},$$

Combining these shifts gives us expressions for the shifted nuclear-spin transition frequencies, for both ^14^N-*V* and ^15^N-*V*:

(C6)
f1=|Q|+γ14nBz−A⊥2F−−A⊥2(|Q|−|A‖|F−2+2|Q|−|A‖|F+2)+γe2Bx22[A⊥23Q(1F++1F−)2−|A‖|(1F−2−1F+2)],f2=|Q|−γ14nBz−A⊥2F+−A⊥2(2|Q|−|A‖|F−2+|Q|−|A‖|F+2)+γe2Bx22[A⊥23Q(1F++1F−)2+|A‖|(1F−2−1F+2)],f3=|Q|−|A‖|+γ14nBz−A⊥2(2|Q|−|A‖|F−2)+γe2Bx22[A⊥2(2Q−A‖+1Q+A‖)1F−2+|A‖|F−2],f4=|Q|+|A‖|−γ14nBz+A⊥2F−−A⊥2(|Q|F−2)+γe2Bx22[A⊥2(1Q−A‖+2Q+A‖)1F−2−|A‖|F−2],f5=|Q|+|A‖|+γ14nBz+A⊥2F+−A⊥2(|Q|F+2)+γe2Bx22[A⊥2(1Q−A‖+2Q+A‖)1F+2−|A‖|F+2],f6=|Q|−|A‖|−γ14nBz−A⊥2(2|Q|−|A‖|F+2)+γe2Bx22[A⊥2(2Q−A‖+1Q+A‖)1F+2+|A‖|F+2],

and

(C7)
f7=|γ15n|Bz+A⊥22(1F−−1F+)+A⊥24(A‖F−2−A‖F+2)+γe2Bx22[A⊥2|15γn|Bz(1F++1F−)2−A‖(1F−2−1F+2)],f8=A‖−|γ15n|Bz−A⊥22(1F−)−A⊥24(A‖F−2)+γe2Bx22[(A⊥2A‖−A‖)1F−2],f9=A‖+|γ15n|Bz−A⊥22(1F+)−A⊥24(A‖F+2)+γe2Bx22[(A⊥2A‖−A‖)1F+2].

These expressions can be reduced to obtain simplified expressions for the transition frequencies [see Eqs. (2) and (3)], as well as their angular dependences [see Eqs. (7) and (8)].

For ^14^N-*V*, we can linearly combine nuclear-spin transition frequencies in order to obtain simple approximations for γ14nB,Q, and *A*_‖_:

(C8)
γ14nB≈f3−f62,|Q|−|A‖|≈f3+f62,|Q|≈f1+f2+f3+f4+f5+f66,|A‖|≈f1+f2−2f3+f4+f5−2f66.
